# Qualitätssicherungssystem zur Bewertung eines HNO-Facharztrepetitoriums

**DOI:** 10.1007/s00106-021-01065-6

**Published:** 2021-06-09

**Authors:** Tobias Albrecht, Tanja Hildenbrand, Jan Beneke, Christian Offergeld, Wolf Ramackers

**Affiliations:** 1 Hals‑, Nasen- und Ohrenklinik, Universitätsklinikum Heidelberg, Im Neuenheimer Feld 400, 69120 Heidelberg, Deutschland; 2 Hals-, Nasen-, und Ohrenklinik, Universitätsklinikum Freiburg, Freiburg, Deutschland; 3 Klinik für Herz‑, Thorax‑, Transplantations- und Gefäßchirurgie, Medizinische Hochschule Hannover, Hannover, Deutschland; 4 Klinik für Allgemein‑, Viszeral- und Transplantationschirurgie, Medizinische Hochschule Hannover, Hannover, Deutschland

**Keywords:** Wissensmanagement, Assistenzzeit und Facharztausbildung, Facharztweiterbildung, Evaluation, Lernen, Knowledge management, Internship and residency, Medical graduate education, Evaluation, Learning

## Abstract

**Hintergrund:**

Die Facharztweiterbildung ist häufig durch lokal geprägte Weiterbildungsschwerpunkte bestimmt, was zu einem heterogenen Weiterbildungsergebnis führen kann. Repetitorien vor Facharztprüfungen könnten dies harmonisieren.

**Ziel der Arbeit:**

Ziel ist die Darstellung eines Qualitätssicherungssystems zur Bewertung eines Repetitoriums für HNO-Facharztkandidaten.

**Material und Methoden:**

Die Lehreinheiten eines in Präsenz durchgeführten Facharztrepetitoriums wurde mittels Fragebogen evaluiert. Sowohl eine deskriptive Auswertung als auch eine multivariable binär-logistische Regressionsanalyse wurden durchgeführt. Zur Evaluation der Faktoren, die zu einer negativen Wahrnehmung einer Lehreinheit führen, erfolgte eine Fokussierung auf die schlechtesten 15 % aller Gesamtbewertungen. Für ein individuelles Dozentenfeedback wurde exemplarisch ein Stärken-Schwächen-Profil eines Dozenten erstellt.

**Ergebnisse:**

Die Auswertung der Evaluationsergebnisse zeigte eine durchschnittlich sehr gute Gesamtbewertung von 12,8 (±2,4) bei maximal 15 möglichen Punkten. Die multivariable Regression bestimmte die Items „Freundlichkeit“, „Systematischer Aufbau“, „Eigene Mitarbeit“, „Vorwissen“ und „Unterrichtseinheit effizient“ als maßgeblich für eine Negativwahrnehmung einer Lehreinheit. Anhand des Dozentenprofils lassen sich in einer objektiven Form die Stärken und Schwächen des individuellen Dozenten aufzeigen.

**Schlussfolgerung:**

Der entwickelte Fragebogen bildet eine gute Möglichkeit zur Qualitätssicherung einer Lehrveranstaltung in der Weiterbildung. Diese erfolgt zum einen über die Regressionsanalyse aller Fragebögen, zum anderen über die Erstellung eines individuellen Dozentenprofils, welches eine objektive Grundlage zur Verbesserung der einzelnen Lehreinheit durch ein detailliertes Feedback an den Dozenten ermöglicht.

**Zusatzmaterial online:**

Die Online-Version dieses Beitrags (10.1007/s00106-021-01065-6) enthält den Studienfragebogen. Beitrag und Zusatzmaterial stehen Ihnen auf www.springermedizin.de zur Verfügung. Bitte geben Sie dort den Beitragstitel in die Suche ein, das Zusatzmaterial finden Sie beim Beitrag unter „Ergänzende Inhalte“.

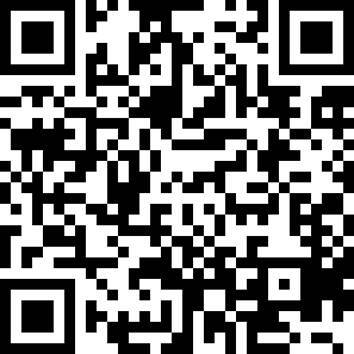

## Abstract (en)

**Background:**

Residency training is often characterized by locally influenced training content and focus, which can lead to heterogeneous training outcomes. Refresher courses before the speciality certificate examinations can harmonize the situation.

**Objective:**

The current publication aims to present a quality management system for evaluation of a postgraduate refresher course for otolaryngology residents.

**Materials and methods:**

The teaching sessions of a postgraduate course were evaluated using questionnaires. Descriptive statistics and multivariable binary logistic regression analysis were performed. To evaluate the factors leading to a negative perception of a teaching session, the focus was set on the worst 15% of all total ratings. An exemplary strength/weakness profile of a lecturer was created for individual feedback.

**Results:**

Analysis of the evaluation results showed an overall average rating of 12.8 (±2.4) out of a maximum of 15 possible points. Multivariable regression determined the items “friendliness,” “systematic structure,” “own involvement,” “prior knowledge,” and “efficient teaching session” to be significant for a negative perception of a teaching session. Using the lecturer profile, the strengths and weaknesses of the individual lecturer can be shown in an objective manner.

**Conclusion:**

The developed questionnaire represents a good tool for quality management of a postgraduate refresher course for otolaryngology residents. This is achieved by regression analysis and creation of an individual lecturer profile, which provides an objective basis for improving the individual teaching session through detailed feedback to the lecturer.

Die Hals-Nasen-Ohren-Heilkunde wird traditionell von Medizinstudierenden wie auch vielen Medizinern als eines der „kleinen Fächer“ angesehen. Vor diesem Hintergrund scheint die Weiterbildungszeit von 60 Monaten für den Facharzt/die Fachärztin für Hals-Nasen-Ohren-Heilkunde auf den ersten Blick ein vergleichsweise langes Zeitintervall [1]. Im Rahmen der Weiterbildung muss jedoch nicht nur der Umgang mit Patienten aller Altersklassen und Geschlechter erlernt werden, sondern auch eine Vielzahl diagnostischer Möglichkeiten und zahlreiche konservative Therapieoptionen. Darüber hinaus gehören qualitativ anspruchsvolle chirurgische Eingriffe zum Repertoire von HNO-Fachärzt(inn)en. Gerade aber das hohe Maß an diagnostischer und therapeutischer Diversität und eine hohe Erwartungshaltung von Patientenseite in Bezug auf einen Therapieerfolg können sich einerseits als Hemmschuh, andererseits aber auch als Katalysator in der Umsetzung einer strukturierten Weiterbildung erweisen. Neben der immer dominanter werdenden betriebswirtschaftlichen Komponente können der Personalschlüssel der Kliniken und Unterschiede im Behandlungsspektrum zwischen Universitätskliniken und Kliniken in städtischer, kirchlicher oder privater Trägerschaft einen weiteren Einflussfaktor darstellen [2].

In der Weiterbildungsordnung in Deutschland existiert, anders als beispielsweise in der schweizerischen HNO-Gesellschaft, keine festgeschriebene Rotation oder ein Austausch von Weiterbildungsassistent(inn)en zwischen verschiedenen Einrichtungen [1, 3]. Daher wird die Weiterbildung der Assistent(inn)en, entsprechend den Behandlungsschwerpunkten der Klinik, häufig durch lokal geprägte Weiterbildungsinhalte bestimmt. Dies betrifft sowohl das theoretische und diagnostische Vorgehen wie auch die therapeutischen Optionen. Um sich mit Inhalten außerhalb des Schwerpunkts der Ausbildungsklinik vertraut zu machen, ist ein hohes Maß an Motivation mit eigenverantwortlichem Studium von Lehrbüchern, Leitlinien oder Publikationen, die Teilnahme an gebührenpflichtigen Fortbildungen oder ein Arbeitsplatzwechsel notwendig [4].

Diese Inkongruenzen in der lokalen Weiterbildung kumulieren in unterschiedliche Erwartungs- und Anspruchshaltungen der Assistenten an kompakte Lehrformate zur Facharztvorbereitung wie z. B. Repetitorien [2]. Dieses Lehrformat soll in erster Linie einen großen Umfang an Lehrstoff kompakt, übersichtlich und in kurzer Zeitdauer präsentieren. Im Idealfall sollte eine derartige Veranstaltung jeden der Teilnehmer aus einer Gruppe mit heterogenem Vorwissen an seiner individuellen Position abholen und zum Ende der Veranstaltung den Wissensstand in der Gruppe weitestgehend nivellieren [5, 6]. Dieses Ziel ist in einer kompakten Veranstaltung wie einem Repetitorium nur unter Umsetzung einer entsprechenden medizindidaktischen Konzeption und mittels inkludierter Qualitätskontrolle erreichbar. Mit einer permanenten Qualitätskontrolle können die globale Veranstaltung wie auch die einzelnen Beiträge kritisch hinterfragt, eingeordnet und kontinuierlich zielgruppenorientiert verbessert werden [7]. Nur durch ein Feedback der Teilnehmer ist für Organisatoren und für die aus unterschiedlichen Einrichtungen stammenden Dozenten und Dozentinnen eine realistische Einschätzung der Gesamtveranstaltung wie auch der Teilbereiche zur Wissensvermittlung, Niveauregulation und Integration in die Weiterbildung möglich [8, 9].

Ziel dieser Arbeit ist die Darstellung eines Qualitätssicherungssystems mit dessen Einzelkomponenten zur Bewertung eines Repetitoriums für HNO-Facharztkandidaten.

## Studiendesign und Untersuchungsmethoden

Evaluiert wurde ein 3‑tägiges Facharztrepetitorium, welches mit 40 Teilnehmern in Präsenz stattfand. Die Lehrinhalte wurden in Seminarform von insgesamt 20 Dozenten vermittelt. Direkt nach jeder Lehreinheit wurde diese durch die Teilnehmer mittels Fragebogen evaluiert. Der Fragebogen (s. elektronisches Zusatzmaterial online) umfasst spezifische Items zum Dozenten, zur Lehreinheit und zur Selbstwahrnehmung der Teilnehmer. Zusätzlich wurde der subjektive Lernzuwachs durch Einschätzung des eigenen Wissens vor und nach der Unterrichtseinheit ermittelt. Die Fragebogenitems wurden mithilfe einer Likert-Skala nach dem Schulnotensystem (1 = sehr gut bzw. trifft voll zu, 6 = ungenügend bzw. trifft überhaupt nicht zu) bewertet. Abschließend erfolgte eine globale Bewertung der Unterrichtseinheit nach dem gymnasialen Oberstufensystem von 0 bis 15 Punkte (15 Punkte = sehr gut; 0 Punkte = ungenügend). Die Werte der Fragebogenitems wurden in ordinalen Werten erfasst. Eine deskriptive Beschreibung erfolgte sowohl über Mittelwert und Standardabweichung als auch über Median mit Interquartilsabstand. Binäre Daten wurden über Gesamtzahl und Prozentwert am jeweiligen Kollektiv abgebildet. Unter der Annahme, dass fehlende Werte aus Sicht der Teilnehmenden eine nicht vorhandene Eigenschaft darstellten, die somit auch nicht bewertet werden konnte, wurden bei fehlenden Werten für Variablen die jeweils schlechteste Bewertung angenommen und der Zahlenwert 6 eingesetzt [10]. Ausgenommen von der Imputation waren die Variablen „Wissen vorher“, „Wissen nachher“ und „Wissensdifferenz“.

Für die vergleichenden Statistiken wurden die ordinalen Variablen zunächst mittels Kolmogorov-Smirnov- und Shapiro-Wilk-Test auf Normalverteilung geprüft. Bei Signifikanz einer der beiden Tests wurde eine nichtparametrische Verteilung angenommen. Die statistischen Gruppenvergleiche erfolgten für nichtparametrische Daten mit Wilcoxon-Rang-Summentest. Die dichotome Variable „Negativwahrnehmung“ wurde durch die schlechtesten 15 % der Bewertungen nach Gesamtnote definiert.

Die Ermittlung der Einflussgrößen auf den dichotomen Endpunkt „Negativwahrnehmung“ erfolgte zunächst mittels einer univariablen sowie nachfolgend mit einer multivariablen binären logistischen Regression. Die Ergebnisse sind entsprechend als Odds-Ratio-Schätzer und 95%-Konfidenzgrenzen dargestellt. Für das Rückwärtsverfahren („backward likelihood“), das als Teil der multivariablen Regressionsanalyse durchgeführt wurde, sind ergänzend die „receiver operating characteristics curves“ (ROC-Kurven) berechnet. Die Auswertung erfolgte mit SAS 9.4 Enterprise Guide (SAS Institute, Cary, NC, USA).

## Ergebnisse

Von den insgesamt 800 ausgegebenen Fragebögen wurden 517 Fragebögen ausgefüllt zurückgegeben, was einer Rücklaufquote von 64,6 % entspricht. Wegen einer fehlenden Endnote mussten 23 Fragebögen ausgeschlossen werden, sodass 494 Fragebögen in die Auswertung eingeschlossen werden konnten. Die deskriptive Statistik mit Mittelwert und Standardabweichung bzw. Median mit Interquartilsabstand für das Gesamtkollektiv aller Lehrveranstaltungen und aller Fragebogenitems ist in Tab. 1 dargestellt. Inhaltlich wurden die Items einer der 3 Kategorien Dozent, Teilnehmer und Struktur zugeordnet.

**Table 1:** 

Fragebogenitem	Mittelwert (± SD)		Median (IQR)
*Dozent*			
Stoffpräsentation	1,1	(±0,4)	1 (1–1)
Aufmerksamkeit erhalten	1,7	(±4,5)	1 (1–2)
Mitarbeit gefördert	1,3	(±0,8)	1 (1–1)
Anschauliche Beispiele	1,3	(±0,8)	1 (1–1)
Verständliche Erklärungen	1,6	(±1)	1 (1–2)
Freundlichkeit	1,1	(±0,4)	1 (1–1)
Pünktlichkeit	1	(±0,2)	1 (1–1)
Tempo zu hoch	4,4	(±1,8)	5 (3–6)
*Teilnehmer*			
Eigene Mitarbeit	1,8	(±1,2)	1 (1–2)
Vorwissen	1,9	(±1,3)	1 (1–2)
Inhalt interessant	1,6	(±1,1)	1 (1–2)
Inhalt relevant	1,7	(±1,2)	1 (1–2)
Unterrichtseinheit effizient	2	(±1,3)	1 (1–3)
*Struktur*			
Systematischer Aufbau	1,4	(±0,8)	1 (1–2)
Lernziele genannt	1,8	(±1,3)	1 (1–2)
Niveau zu hoch	4,4	(±1,8)	5 (3–6)
*Wissensstand*			
Wissen vorher	2,7	(±1,2)	2 (2–3)
Wissen nachher	2,1	(±0,9)	2 (1–2)
Wissenszuwachs	0,7	(±0,8)	1 (0–1)
Gesamtnote	12,8	(±2,4)	14 (12–15)

*IQR* Interquartilsabstand („interquartile range“), *SD* Standardabweichung („standard deviation“)

**Figure 1:**
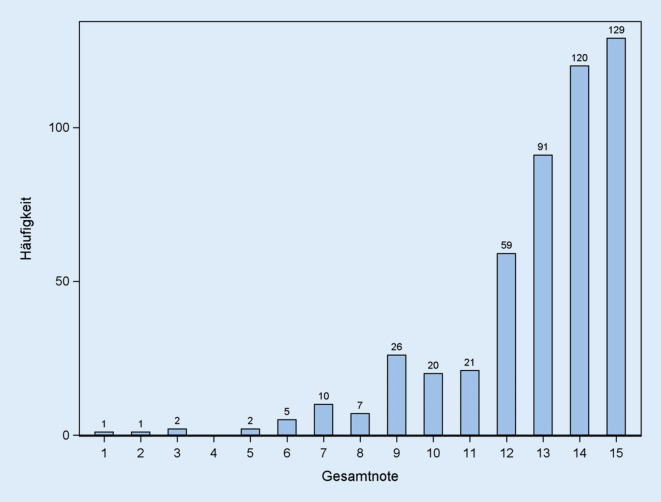

Die globale Bewertung der Unterrichtseinheit analog zum deutschen Oberstufensystem zeigte eine deutliche Rechtsverschiebung mit einem Mittelwert von 12,8 ± 2,4 Punkten (Abb. 1). Von der Gesamtnote ausgehend wurden die 15 % schlechtesten Bewertungen ermittelt. Diese wurden als eine negative Wahrnehmung der einzelnen Unterrichtseinheit definiert und lagen bei einer Gesamtnote von 10 oder weniger Punkten. Die anderen 85 % der Fragebögen wurden als eine positive Wahrnehmung der Unterrichtseinheit definiert.

Der deskriptive Vergleich zwischen der Gruppe mit einer positiven Wahrnehmung und einer negativen Wahrnehmung der jeweiligen Unterrichtseinheit ergab in allen Fragebogenitems bis auf die Pünktlichkeit (*p* = 0,6602) signifikante Unterschiede. Abgesehen von der Frage nach einem zu hohen Tempo (*p* = 0,0028) waren diese sogar hochsignifikant (*p* < 0,0001; Tab. 2).

**Table 2:** 

Variable	Negativwahrnehmung		Positivwahrnehmung		
	*n* = 74		*n* = 420		
	Mittelwert (± SD)	Median (IQR)	Mittelwert (± SD)	Median (IQR)	*p-*Wert
*Dozent*					
Stoffpräsentation	1,216 (±0,414)	1 (1–1)	1,071 (±0,352)	1 (1–1)	< 0,0001
Aufmerksamkeit erhalten	3,068 (±1,358)	3 (2–4)	1,474 (±4,801)	1 (1–1)	< 0,0001
Mitarbeit gefördert	2,027 (±1,334)	2 (1–2)	1,188 (±0,607)	1 (1–1)	< 0,0001
Anschauliche Beispiele	2,081 (±1,03)	2 (1–3)	1,181 (±0,599)	1 (1–1)	< 0,0001
Verständliche Erklärungen	2,77 (±1,319)	3 (2–3)	1,369 (±0,697)	1 (1–2)	< 0,0001
Freundlichkeit	1,392 (±0,615)	1 (1–2)	1,045 (±0,302)	1 (1–1)	< 0,0001
Pünktlichkeit	1,027 (±0,163)	1 (1–1)	1,029 (±0,238)	1 (1–1)	0,6602
Tempo zu hoch	3,851 (±1,841)	4 (2–6)	4,505 (±1,733)	5 (3–6)	0,0028
*Teilnehmer*					
Eigene Mitarbeit	3,365 (±1,486)	3 (2–5)	1,469 (±0,836)	1 (1–2)	< 0,0001
Vorwissen	3,405 (±1,604)	4 (2–5)	1,595 (±0,951)	1 (1–2)	< 0,0001
Inhalt interessant	2,973 (±1,489)	3 (2–4)	1,338 (±0,725)	1 (1–1)	< 0,0001
Inhalt relevant	3,176 (±1,683)	3 (2–5)	1,455 (±0,947)	1 (1–2)	< 0,0001
Unterrichtseinheit effizient	3,689 (±1,489)	4 (3–5)	1,669 (±1,049)	1 (1–2)	< 0,0001
*Struktur*					
Systematischer Aufbau	2,392 (±1,291)	2 (1–3)	1,226 (±0,511)	1 (1–1)	< 0,0001
Lernziele genannt	3,338 (±1,777)	3 (2–5)	1,583 (±1,046)	1 (1–2)	< 0,0001
Niveau zu hoch	3,649 (±1,708)	3 (2–5)	4,533 (±1,763)	5 (3–6)	< 0,0001

Als Negativwahrnehmung wurde die schlechtesten 15 % der Gesamtbewertungen definiert

*IQR* Interquartilsabstand („interquartile range“), *SD* Standardabweichung („standard deviation“)

Die weitergehende Analyse wurde mittels multivariabler binär-logistischer Regression durchgeführt. Durch eine schrittweise Regression mit Anwendung des Rückwärtsverfahren (Backward-Eliminations-Methode) konnten die Variablen bestimmt werden, die maßgeblich zu einer negativen Wahrnehmung der Lehreinheit führen. Dies waren die Fragebogenitems „Freundlichkeit“, „Systematischer Aufbau“, „Eigene Mitarbeit“, „Vorwissen“ und „Unterrichtseinheit effizient“ (Tab. 3). Die AUROC-Analyse („area under the receiver operating characteristic“) als Maß für die diagnostische Güte ergab 0,929.

**Table 3:** 

Fragebogenitem	Odds-Ratio	95%-Wald-Konfidenzgrenzen	
Freundlichkeit	2,392	1,345	4,256
Systematischer Aufbau	2,181	1,402	3,391
Eigene Mitarbeit	1,554	1,091	2,214
Vorwissen	1,39	1,04	1,858
Unterrichtseinheit effizient	1,826	1,416	2,354

Die aufgeführten Items tragen maßgeblich zu einer Negativwahrnehmung einer Lehreinheit bei.

Der Einfluss des einzelnen Items kann anhand der Odds-Radio bestimmt werden

Für das individuelle Dozentenfeedback wurden die Werte des einzelnen Dozenten einem Vergleichskollektiv, bestehend aus allen anderen Dozenten mit einer guten Bewertung, gegenübergestellt und auf signifikante Unterschiede überprüft. Exemplarisch zeigt Tab. 4 die Mittelwerte und den Median der 23 Fragebögen eines Dozenten im Vergleich zu der Gruppe der gut bewerteten Dozenten.

**Table 4:** 

Variable	Mittelwert (± SD)		Median (IQR)		*p*-Wert
	Dozent (*n* = 23)	Vergleichsgruppe (*n* = 380)	Dozent (*n* = 23)	Vergleichsgruppe (*n* = 380)	
*Dozent*					
Stoffpräsentation	1,12 (±0,33)	1,06 (±0,34)	1 (1–1)	1 (1–1)	0,167
Aufmerksamkeit erhalten	2,75 (±0,391)	1,52 (±5,05)	3 (2–3,5)	1 (1–1)	< 0,0001
Mitarbeit gefördert	1,91 (±1,13)	1,16 (±0,51)	1 (1–3)	1 (1–1)	< 0,0001
Anschauliche Beispiele	2,04 (±1,08)	1,17 (±0,56)	2 (1–3)	1 (1–1)	< 0,0001
Verständliche Erklärungen	2,45 (±1,17)	1,36 (±0,65)	2 (1,5–3)	1 (1–2)	< 0,0001
Freundlichkeit	1,25 (±0,608)	1,06 (±0,33)	1 (1–1)	1 (1–1)	0,014
Pünktlichkeit	1,0 (±0)	1,02 (±0,19)	1 (1–1)	1 (1–1)	0,539
Tempo zu hoch	2,87 (±1,75)	2,43 (±1,68)	2 (1,5–5)	2 (1–4)	0,131
*Teilnehmer*					
Eigene Mitarbeit	3,16 (±1,57)	1,42 (±0,77)	3 (2–4,5)	1 (1–2)	< 0,0001
Vorwissen	3,58 (±1,58)	1,52 (±0,86)	4 (2–5)	1 (1–2)	< 0,0001
Inhalt interessant	3,29 (±1,48)	1,3 (±0,68)	3,5 (2–4)	1 (1–1)	< 0,0001
Inhalt relevant	3,37 (±1,66)	1,39 (±0,84)	3,5 (2–5)	1 (1–2)	< 0,0001
Unterrichtseinheit effizient	2,95 (±1,51)	1,64 (±0,97)	3 (1–4)	1 (1–2)	< 0,0001
*Struktur*					
Systematischer Aufbau	2,04 (±0,95)	1,23 (±0,55)	2 (1–3)	1 (1–1)	< 0,0001
Lernziele genannt	2,95 (±1,45)	1,58 (±1,07)	3 (2–3,5)	1 (1–2)	< 0,0001
Niveau zu hoch	3,62 (±1,68)	2,38 (±1,72)	4 (2–5)	2 (1–3)	0,002

*IQR* Interquartilsabstand („interquartile range“), *SD* Standardabweichung („standard deviation“)

Die Analyse ergab in der Kategorie „Dozent“ bei den Fragebogenitems „Aufmerksamkeit erhalten“, „Mitarbeit gefördert“, „Anschauliche Beispiele“ und „Verständliche Erklärungen“ hochsignifikante (*p* < 0,0001) Unterschiede. In der Kategorie „Teilnehmer“ waren die Items „Eigene Mitarbeit“, „Vorwissen“, „Inhalt interessant“, „Inhalt relevant“ und „Unterrichtseinheit effizient“ hochsignifikant (*p* < 0,0001). In der Kategorie „Struktur“ konnten hochsignifikante (*p* < 0,0001) Unterschiede für die Fragebogenitems „Systematischer Aufbau“ und „Lernziele genannt“ gezeigt werden.

Signifikant mit niedrigerem Signifikanzniveau waren „Freundlichkeit“ (*p* = 0,014) und „Niveau zu hoch“ (*p* = 0,002).

## Diskussion

In der HNO-Heilkunde endet die Weiterbildungszeit regelhaft mit dem Absolvieren einer Facharztprüfung. Hierfür müssen während der Weiterbildungszeit die im Logbuch aufgeführten diagnostischen und operativen Tätigkeiten erfüllt werden [1].

Lernziele bzw. Kompetenzen, die ein einheitliches Ziel der Facharztweiterbildung ermöglichen, werden im Logbuch nicht definiert [1].

Durch eine fehlende Standardisierung der Weiterbildung kann es, da die Facharztweiterbildung maßgeblich durch den Weiterbilder und die Weiterbildungseinrichtung geprägt wird, in unterschiedlichen Einrichtungen zu unterschiedlichen Ausbildungsständen der Kandidat(inn)en vor der Facharztprüfung kommen. Eine fehlende Standardisierung der Facharztprüfung in Deutschland hinsichtlich der Anforderungen und Wissensinhalte, wie sie z. B. in der europäischen Facharztprüfung existiert, verhindert zudem eine assessmentgetriggerte Ausbildung [11]. Ein unabhängiges Facharztrepetitorium kann dazu beitragen, die Prüflinge vor der Facharztprüfung auf ein einheitliches theoretisches Wissensniveau zu bringen. Um diese Aufgabe erfolgreich zu meistern, bedarf es einer zielgruppenorientierten Wissensvermittlung. Hierfür ist die Kenntnis der Bedürfnisse der Zielgruppe eine Schlüsselkomponente [8]. Eine Möglichkeit zur Erfassung der Bedürfnisse ist das Einholen eines Feedbacks nach einer Lehrveranstaltung [9, 12]. Dieses Feedback kann auf verschieden Arten erfolgen. Die Anforderungen an die Art des Feedbacks sollten eine einfache Anwendbarkeit und die Lieferung zuverlässiger Daten mit eindeutigen Informationen im Hinblick auf eine Verbesserung sein [12].

Fragebögen sind eine häufig verwendete Methode, um effizient und routinemäßig Feedback von einer größeren Gruppe zu erhalten [13–15]. Ein Problem von Feedback ist jedoch häufig die Subjektivität [16, 17]. Wenn die Stichprobengröße bei einer Erhebung mittels Fragebogen jedoch groß genug ist, können aus einem subjektiven Feedback die objektiven Informationen extrahiert werden. Eine systematische statistische und objektive Analyse und Interpretationen der Evaluationsdaten unterstützen eine gezielte Verbesserung der evaluierten Veranstaltung [7, 10]. Eine Verbesserung basierend auf Evaluationsdaten kann allerdings auch schwierig sein. Zunächst muss ein Fragebogen entwickelt werden, der die relevanten Fragen beinhaltet, deren Beantwortung zu einer Verbesserung führen [10, 18]. Darüber hinaus erfordern die Daten eine sorgfältige und umfassende Analyse, um Schlussfolgerungen für eine Verbesserung der Veranstaltung zu ermöglichen [10, 19–21]. Mithilfe der durchgeführten Datenanalysen war es möglich, die relevanten Faktoren zu ermitteln, die bei den Teilnehmern eine positive oder negative Wahrnehmung der Veranstaltung hervorrufen. Hierbei waren die Faktoren „Freundlichkeit“, „Systematischer Aufbau“, „Vorwissen“, „Eigene Mitarbeit“ und „Unterrichtseinheit effizient“ maßgeblich. Freundlichkeit ist eine Grundvoraussetzung für die Kommunikation mit einer Zielgruppe und daher ein wichtiges Element.

Durch eine gut strukturierte Lehrveranstaltung fällt es den Teilnehmern leichter, gedanklich zu folgen. Dies bildet die Grundlage, um neue Informationen aufzunehmen („Systematischer Aufbau“) [4, 6, 22]. Die Zielgruppenorientierung zeigt sich an dem Element „Vorwissen“. Um neue Inhalte einzuordnen und abzuspeichern, benötigt der Lernende ein gewisses Maß an Vorwissen [5, 6]. Um das neu zu vermittelnde Wissen an ihr Vorwissen anknüpfen zu können, ist eine Orientierung am meist unterschiedlichen Vorwissen der Teilnehmer wichtig für den Lernerfolg. Hierdurch kann einer Überforderung der Teilnehmer bei komplexen und umfangreichen Inhalten vorgebeugt werden. Dies kann sich natürlich bei einer großen heterogenen Gruppe schwierig gestalten. Die Variable „Eigene Mitarbeit“ ist ein Surrogat für die aktive Mitarbeit der Teilnehmer. Da Lernen ein aktiver Prozess ist, ist die aktive Mitarbeit eine Voraussetzung für den Lernprozess. Je aktiver der Lernende in den Lehrprozess involviert ist, desto größer ist sein Lernzuwachs [23, 24]. Damit die Teilnehmer aktiv mitarbeiten können, muss sich die jeweilige Unterrichtseinheit nicht nur am Vorwissen der Teilnehmer orientieren und den Inhalt gut strukturiert präsentieren, sondern auch aktiv zur Mitarbeit anregen und die Interaktion fördern. Der letzte Punkt „Unterrichtseinheit effizient“ spiegelt die Erwartung der Zielgruppe an das Format eines kompakten Repetitoriums als eine effiziente Wissensvermittlung wider.

Zusätzlich zu einer globalen Auswertung der Veranstaltung wurde eine Subgruppenanalyse nach Dozenten durchgeführt. Für einen dieser Dozenten wurde exemplarisch ein individuelles Profil von Stärken und Schwächen gezeigt. In der exemplarischen Dozentenauswertung zeigte sich in allen 3 Kategorien des Fragebogens „Dozent“, „Teilnehmer“ und „Struktur“ Verbesserungspotenzial.

In der Kategorie Dozent schnitten die Items „Aufmerksamkeit erhalten“, „Mitarbeit gefördert“, „Anschauliche Beispiele“ und „Verständliche Erklärungen“ im Vergleich zu den anderen Dozenten signifikant schlechter ab. Bei den strukturellen Aspekten der Lehrveranstaltung konnten die Variablen „systematischer Aufbau“ und „Lernziele genannt“ im Vergleich zu den anderen Dozenten ebenso als signifikant schlechter identifiziert werden. Darüber hinaus sahen die Teilnehmer die fehlende Möglichkeit zur Mitarbeit als Kritikpunkt.

Die Variablen „Vorwissen“ „Niveau zu hoch“, „Inhalt interessant“ und „Inhalt relevant“ unterschieden sich ebenfalls hochsignifikant vom Vergleichskollektiv. Das Thema schien für die Teilnehmer nicht interessant und wurde als nicht relevant bewertet. Das Niveau wurde im Verhältnis zum Vorwissen von den Teilnehmern als zu hoch eingestuft. Dies ist ein gutes Beispiel für ein schwieriges Thema einer Lehreinheit. Die Konstellation des geringen Vorwissens und der schwer nachvollziehbaren Relevanz des Themas stellt eine Herausforderung für den Dozenten dar. Die Relevanz des Themas zu vermitteln und das Interesse zu wecken, ist für den Lernerfolg der Teilnehmer eine wichtige Voraussetzung [25, 26]. In diesem Fall kam erschwerend hinzu, dass das Niveau der Lehreinheit nicht dem Vorwissen der Teilnehmer angepasst war.

Die Auswertung bildet eine objektive Grundlage für ein Verbesserungsgespräch mit dem Dozierenden, sodass in zukünftigen Lehreinheiten wichtige Aspekte wie z. B. die Struktur der Lehrveranstaltung angepasst und die Lernzielkommunikation erfolgen kann. Anhand der Variablen „Vorwissen“ und „Niveau zu hoch“ lässt sich der Inhalt der Lehreinheit zukünftig besser auf die Teilnehmer abstimmen.

Um eine kompakte Veranstaltung wie ein Facharztrepetitorium so effizient wie möglich zu gestalten, ist eine stetige Qualitätskontrolle unerlässlich. Der eingesetzte Fragebogen ermöglicht ein Feedback sowohl für den Veranstalter über die Gesamtveranstaltung als auch über die eingesetzten Dozenten. Durch das Feedback lassen sich nicht nur die Inhalte der Veranstaltung besser auf die Bedürfnisse der Teilnehmer abstimmen, sondern auch die präsentierten Inhalte in Art und Aufbereitung möglichst lernfördernd darstellen.

Methodenkritisch bleibt anzumerken, dass sich die vorliegende Arbeit auf die Wahrnehmung der Qualität der Unterrichtseinheit durch die Teilnehmer fokussiert. Es verbliebe noch die Möglichkeit der Messung des Lernerfolgs über eine Evaluation des Abschneidens der Teilnehmer in der Facharztprüfung. Hierbei sei aber auf die eingeschränkte Aussagekraft des Prüfungsergebnisses aufgrund einer fehlenden Standardisierung der Facharztprüfungen in Deutschland hingewiesen. Somit verbleibt die Messung der Qualität aus Sicht der Teilnehmer nach Ansicht der Autoren die am besten realisierbare Methode.

## Fazit für die Praxis

- Der vorgestellte Fragebogen ist in Kombination mit den aufgezeigten statistischen Verfahren eine zeit- und ressourcenschonende Möglichkeit für das Qualitätsmanagement des Facharztrepetitoriums als Gesamtveranstaltung sowie der individuellen Beiträge.
- Die Fragebogenitems lassen sich hinsichtlich ihrer Relevanz hierarchisch ordnen und gegeneinander gewichten.
- Durch die Ermittlung der Punkte mit dem größten Effekt auf die Negativwahrnehmung ist eine zielgruppenorientierte und effiziente Verbesserung der Gesamtveranstaltung möglich.
- Die Dozentenanalyse ermöglicht die Erstellung eines individuellen Stärken- und Schwächenprofils der Dozenten, was als eine gute Grundlage für ein individuelles Feedback an jeden Dozenten dienen kann.

## Supplementary Information


