# Supplementary material for: Quality management in a postgraduate refresher course in otolaryngology
Source: HNO. 2021 Jun 9;69(7):568–74. [Article in German] doi: 10.1007/s00106-021-01065-6 (PMC8233240; doi:10.1007/s00106-021-01065-6)
Supplement: Supplementary file 1 [file 106_2021_1065_MOESM1_ESM.pdf]

Repetitorium ORL – Evaluation der Unterrichtseinheit

Der Fragenbogen wurde von der AG Lehrevaluation (MHH) entwickelt und wird in Kooperation mit dem Repetitorium ORL eingesetzt.
Die Auswertung erfolgt selbstverständlich strikt anonym!

Sie können mit Ihren Einschätzungen dazu beitragen, dass die Qualität der Lehre gesteuert und verbessert wird. Bitte füllen Sie deshalb den Fragebogen sorgfältig und vollständig aus. Nur so ist es möglich, aufgrund Ihrer Bewertungen auch kurzfristig Konsequenzen zu ziehen. Ihre Bewertungen können Sie - sofern nicht anders angegeben - auf den vorgegebenen Skalen abstufen.

Name der/s Dozentin/Dozenten: Datum:

**Die Dozentin / Der Dozent**	Trifft voll zu					Trifft gar nicht zu
… war pünktlich anwesend.	☐	☐	☐	☐	☐	☐
… präsentierte den Stoff sicher.	☐	☐	☐	☐	☐	☐
… verwendete anschauliche Beispiele.	☐	☐	☐	☐	☐	☐
… hielt die Aufmerksamkeit durch seinen/ihren Stil.	☐	☐	☐	☐	☐	☐
… begrüßte die Mitarbeit/Nachfragen der Teilnehmer.	☐	☐	☐	☐	☐	☐
… war freundlich.	☐	☐	☐	☐	☐	☐
… hatte ein zu hohes Tempo.	☐	☐	☐	☐	☐	☐
… erklärte komplexe Sachverhalte verständlich.	☐	☐	☐	☐	☐	☐
**Allgemeine Fragen:**						
Es wurden konkrete Lernziele für diese Unterrichtseinheit genannt.	☐	☐	☐	☐	☐	☐
Die Unterrichtseinheit hatte einen systematischen Aufbau.	☐	☐	☐	☐	☐	☐
Ich konnte gut mitarbeiten.	☐	☐	☐	☐	☐	☐
Mit meinem Vorwissen konnte ich inhaltlich gut folgen.	☐	☐	☐	☐	☐	☐
Das Niveau dieser Unterrichtseinheit war zu hoch.	☐	☐	☐	☐	☐	☐
Der Inhalt dieser Unterrichtseinheit ist für mich relevant.	☐	☐	☐	☐	☐	☐

Bitte füllen Sie auch die zweite Seite aus!

Allgemeine Fragen:

	Trifft voll zu					Trifft überhaupt
Der Inhalt dieser Unterrichtseinheit hat mich interessiert.	☐	☐	☐	☐	☐	☐
Diese Unterrichtseinheit war verglichen mit dem Eigenstudium effizient.	☐	☐	☐	☐	☐	☐

Wissen:

	sehr gut	gut	befriedigend	ausreichend	mangelhaft	ungenügend
Wie würden Sie Ihr Wissen VOR der Unterrichtseinheit einstufen?	☐	☐	☐	☐	☐	☐
Wie würden Sie Ihr Wissen NACH der Unterrichtseinheit einstufen?	☐	☐	☐	☐	☐	☐

Globale Bewertung der Unterrichtseinheit:

Wie beurteilen Sie das Seminar insgesamt? (0 Pkt. = ungenügend 3 Pkt. = mangelhaft 6 Pkt. = ausreichend 9 Pkt.= befriedigend 12 Pkt. = gut 15 Pkt. = sehr gut)

0	1	2	3	4	5	6	7	8	9	10	11	12	13	14	15
☐	☐	☐	☐	☐	☐	☐	☐	☐	☐	☐	☐	☐	☐	☐	☐

Weitere Anmerkungen (Lob und Kritik):

____

____

____

Der ***persönliche Code*** *ermöglicht eine anonyme Zuordnung zu anderen Befragungen. Der (maschinenlesbare) Code setzt sich aus Daten zusammen, die nur Sie kennen und von Ihnen jederzeit wieder gebildet werden können: Zuerst der Anfangsbuchstabe des Vornamens Ihrer Mutter (z.B. Karin = K), dann der Geburtsmonat Ihrer Mutter als Zahl (z.B. März = 3), danach die gleichen Angaben für Ihren Vater, schließlich Ihr Geschlecht (W = weiblich, M = männlich), Ihr Geburtsjahr (2stellig, z.B. 1980 = 80) und das Weiterbildungsjahr (2stellig, z.B. 4. Jahr = 04).*

Anfangsbuchstabe Vorname Mutter	Geburtsmonat Mutter	Anfangsbuchstabe Vorname Vater	Geburtsmonat Vater	Ihr Geschlecht	Ihr Geburtsjahr 2stellig	Weiterbildungsjahr 2stellig
☐	☐☐	☐	☐☐	☐	☐☐	☐☐

Vielen Dank für Ihre Rückmeldung!
